# Liquid biopsy in head and neck cancer patients: blood, saliva, or both ?

**DOI:** 10.1093/pcmedi/pbaf005

**Published:** 2025-02-28

**Authors:** Valentina De Pascale, Federica Ganci, Fabrizio Leone, Valentina Manciocco, Flaminia Campo, Anastasia Mercurio, Alina Catalina Palcau, Claudio Moretti, Frauke Goeman, Sara Donzelli, Giulia Orlandi, Federica Orrù, Renato Covello, Paola Muti, Sabrina Strano, Antonello Vidiri, Raul Pellini, Giovanni Blandino

**Affiliations:** Department of Research and Advanced Technologies, Translational Oncology Research Unit, IRCCS Regina Elena National Cancer Institute, Rome 00144, Italy; Department of Research and Advanced Technologies, Translational Oncology Research Unit, IRCCS Regina Elena National Cancer Institute, Rome 00144, Italy; Department of Research and Advanced Technologies, Translational Oncology Research Unit, IRCCS Regina Elena National Cancer Institute, Rome 00144, Italy; Department of Clinical and Oncology Research, Otolaryngology-Head and Neck Surgery Unit, IRCCS Regina Elena National Cancer Institute, Rome 00144, Italy; Department of Clinical and Oncology Research, Otolaryngology-Head and Neck Surgery Unit, IRCCS Regina Elena National Cancer Institute, Rome 00144, Italy; Department of Research and Advanced Technologies, Pathology Unit, IRCCS Regina Elena National Cancer Institute, Rome 00144, Italy; Department of Clinical and Dermatological Research, Microbiology and Virology Unit, IRCSS San Gallicano Dermatological Institute, Rome 00144, Italy; Department of Clinical and Oncology Research, Otolaryngology-Head and Neck Surgery Unit, IRCCS Regina Elena National Cancer Institute, Rome 00144, Italy; Department of Research and Advanced Technologies, SAFU Unit, IRCCS Regina Elena National Cancer Institute, Rome 00144, Italy; Department of Research and Advanced Technologies, Translational Oncology Research Unit, IRCCS Regina Elena National Cancer Institute, Rome 00144, Italy; Department of Clinical and Dermatological Research, Microbiology and Virology Unit, IRCSS San Gallicano Dermatological Institute, Rome 00144, Italy; Department of Experimental Medicine, Pathology Unit, Sant'Andrea University Hospital, Sapienza University, Rome 00189, Italy; Department of Research and Advanced Technologies, Pathology Unit, IRCCS Regina Elena National Cancer Institute, Rome 00144, Italy; Department of Biomedical, Surgical and Dental Sciences, University of Milan, Milan 20122, Italy; Department of Research and Advanced Technologies, SAFU Unit, IRCCS Regina Elena National Cancer Institute, Rome 00144, Italy; Department of Radiology and Diagnostic Imaging, IRCCS Regina Elena National Cancer Institute, Rome 00144, Italy; Department of Clinical and Oncology Research, Otolaryngology-Head and Neck Surgery Unit, IRCCS Regina Elena National Cancer Institute, Rome 00144, Italy; Department of Research and Advanced Technologies, Translational Oncology Research Unit, IRCCS Regina Elena National Cancer Institute, Rome 00144, Italy

Dear Editor,

Head and neck squamous cell carcinoma (HNSCC) is the sixth most common cancer worldwide, with a constantly growing incidence. HNSCC comprises a group of heterogeneous tumors that originate from the upper digestive region. Tobacco, alcohol abuse, and human papillomaviruses (HPV) infection are the major etiologic agents for HNSCC. Local relapse and metastasis are major causes of mortality, accounting for 50% of cases. Distant metastasis of head and neck cancers are present in ∼10% of HNSCC patients. Diagnosis of distant metastasis is assoc

iated with unfavourable prognosis, with a median survival of ∼10 months [1]. Despite recent advances, multimodality approaches are still not effective in eradicating HNSCC. Liquid biopsy has recently emerged as a very powerful tool that has re-shaped the management of cancer patients worldwide [2–4]. HNSCC patient-derived nucleic acids can be monitored in biological fluids such as blood and saliva, thus suggesting liquid biopsy–molecular approaches as unique tools to refine diagnostic evaluation and therapy allocation. Through minimally invasive procedures, liquid biopsy allows early diagnosis, detection of minimal residual disease, and real-time monitoring of recurrence, metastases, or therapeutic responses [2, 5]. Indeed, the identification and the tracking over time of tumor aberrations released into the body fluids may enable the discovery of novel, non-invasive prognostic and diagnostic biomarkers that can provide an early prediction of disease progression in a timely and cost-effective fashion [6]. Timely prediction of relapse in HNSCC patients is a major challenge that might improve patient survival unprecedently. Liquid biopsy, being only minimally invasive, could be applied to serially monitor surgically resected HNSCC patients in a timely manner to anticipate either relapse or distant metastasis. Furthermore, in the adjuvant setting for HNSCC patients the use of liquid biopsy to monitor the response to treatment will be a unique non-invasive and safe tool with important implications for patients with poor quality of life.

*TP53* mutation is the most frequent genetic alteration (70%–80%) of HPV-negative HNSCC patients. MicroRNAs (miRNAs) are small non-coding and highly stable RNA molecules that are easily detectable in the body fluids of a wide range of cancer patients including those with HNSCC. Precise and serial quantification of circulating analytes in body fluids is a critical step toward clinical application of liquid biopsy in cancer patient management. Digital PCR (dPCR), with its high precision and sensitivity, is the most promising technology for this purpose to accurately detect and quantify rare DNA and RNA sequences in the presence of abundant targets [7].

**Figure 1.**
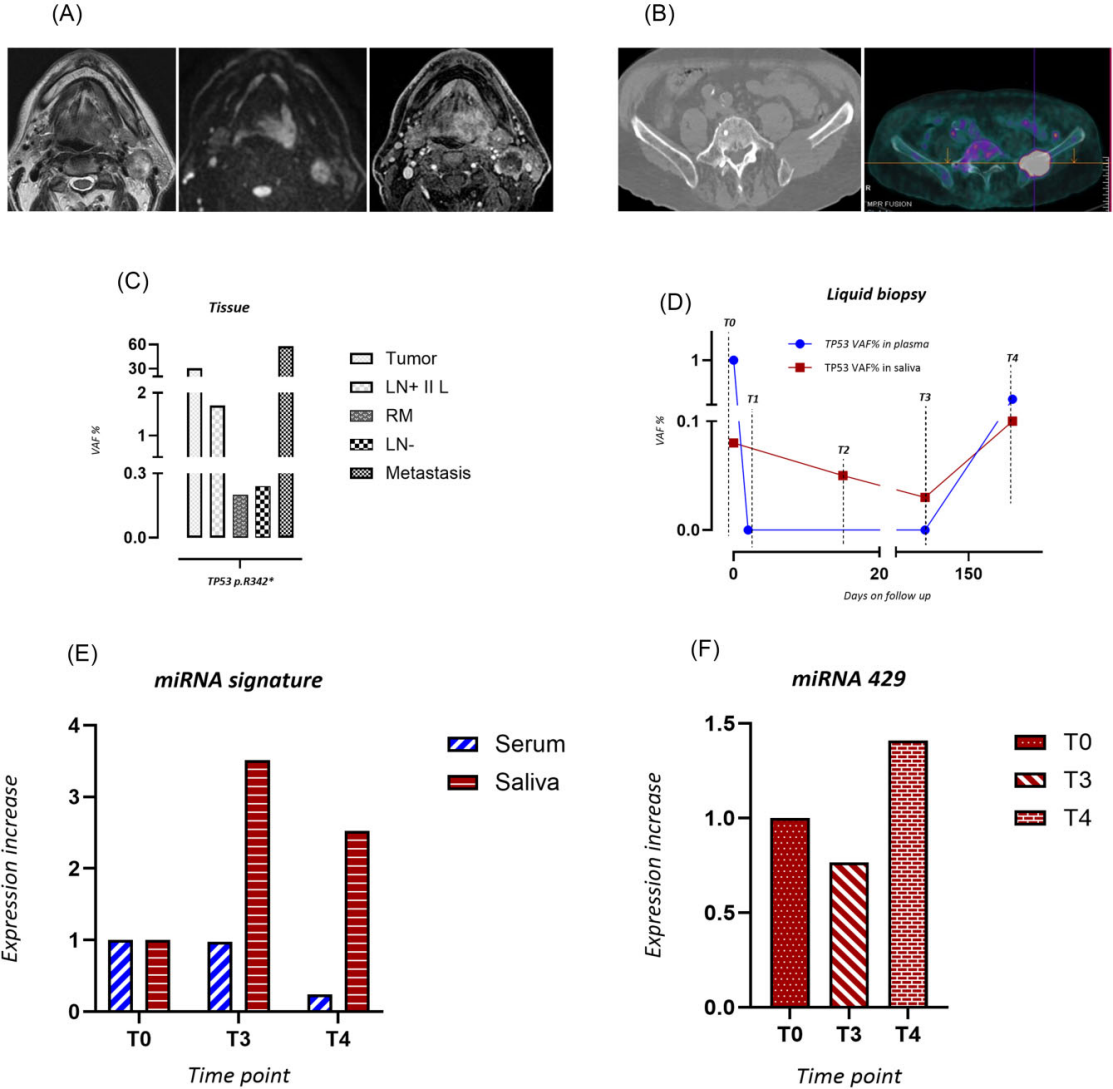
Clinical features and molecular profiling of a metastatic HNSCC patient. (**A**) MRI pre-surgery. T2 sequence, diffusion, and T1 after contrast medium showed the presence of the SCC in the left base of the tongue. (**B**) Imaging techniques for metastatic lesion. CT and PET-CT with FDG after the end of treatment showed the presence of litic metastases into the left iliac wing. (**C**) *TP53* status of HNSCC tissues by dPCR. *TP53* p. R342* and its specific VAF for each collected and analyzed sample tissues are shown. (**D**) Circulating *TP53* status in ctDNA from both plasma and saliva by dPCR. *TP53* p. R342* with specific VAF for each time is shown. (**E**) miRNA signature profile in liquid biopsy. The expression levels of miR-21–3p, miR-21–5p, and miR-96–5p were assessed in saliva and serum samples taken at the different times. (**F**) Detection of circulating miRNA-429 in saliva samples. miR-429 expression was observed as early as in the post-adjuvant therapy sample (T3), and peaked at 2 months before clinical evidence of metastatic lesion (T4).

In the present study we sought to simultaneously profile *TP53* mutations and miRNA expression in multiple liquid biopsy samples from one metastatic HNSCC patient, thereby tracing the related molecular evolution over time. A 69-year-old Caucasian male, p16 and HPV negative, heavy smoker (30 cigarettes/day for 35 years), non alcohol user was referred to our Institute for a cancer lesion (pT4aN3bG3) of the oropharynx (left base of tongue) (Fig. 1A). The patient underwent subtotal glossectomy and neck dissection and was subjected to adjuvant radiotherapy and systemic treatment with weekly cisplatin. A metastatic lesion at the skeletal level (left iliac wing) was identified 3 months after the end of treatment (Fig. 1B). Pembrolizumab treatment for the metastatic lesion lasted for 1 month as the patient's health condition rapidly deteriorated leading to death. We aimed create a portrait of the patient molecularly at diagnosis and throughout his clinical evolution. To this end, we collected the following patient tissues and body fluids: (i) tissues from the primary tumor, metastatic lymph node, and metastatic lesion at left iliac wing; (ii) representative lymph nodes and resection margins histologically tumor free; (iii) plasma samples collected at different time points [i.e. the day before and after surgery (T0 and T1), 5 days after the end of adjuvant treatment (T3), and during clinical follow-up (T4)]; and (iv) serum and saliva samples collected at pre-surgery (T0), at 5-days post treatment (T3), and during follow-up (T4). To monitor patient molecular evolution, we initially assessed the mutational profile of the primary tumor by whole-exome sequencing (WES) analysis. Most of the pathology units including that at our institute perform Next Generation Sequencing (NGS) approaches using targeted panels with a wide range of genes. While this approach is certainly easier to apply than WES, it may suffer in providing medical oncologists with a limited knowledge of the tumoral mutational landscape of a given cancer patient. This might have an impact not only on first line therapy, but also with regard to resistance and metastatic dissemination, in which any genetic alterations—even undruggable—need to be considered in the design of a specific therapeutic approach. Whole exome sequencing (WES) is getting very close to clinical application for both turnaround time and costs, thereby guiding the implementation of an increasingly precise oncological treatment. Specifically, among the mutated genes, we observed a *TP53* p. R342* mutation (c.1024C > T). Of note, the same *TP53* mutation was also detected in histologically tumor-free tissues, metastatic tissue, and in both saliva and plasma by dPCR analysis (Fig. 1C and D). Unexpectedly, all histologically tumor-free tissues (such as resection margins and negative lymph nodes), previously declared histologically negative for tumor cells, exhibited the presence of *TP53* p. R342*, with a Variant Allele Frequency (VAF) > 1% (Fig. 1C). These findings might suggest either minimal residual disease that was not macroscopically and microscopically evident or the presence of preneoplastic clones predisposed to evolve into cancer cells upon adjuvant therapy. Interestingly, we detected the *TP53* mutation p. R342* in circulating tumor DNA (ctDNA) plasma samples collected before surgery (T0, VAF 1%) and 2 months before clinical evidence of skeletal metastatic lesion development (T4, VAF 0.3%). *TP53* p. R342* was not detected in the plasma collected 1 day after surgery and immediately after the end of radiotherapy, probably due to an effect of surgical and systemic treatments (Fig. 1D). Finally, the presence of *TP53* p. R342* at low VAF was also detected in saliva samples (T0 to T3), while an increased VAF was observed 2 months before the histological diagnosis of metastasis (T4) (Fig. 1D). Based on this evidence, a salivary swab might be a more informative, sensitive, and predictive source to incicate the presence of residual neoplastic cells than plasma. One potential explanation for the higher cancer recurrence predictivity of saliva than plasma might reside in the close anatomical association between saliva and cancerous lesions [8]. Thus, in adopting a liquid biopsy approach, it is important to consider the primary cancer site and to analyze the closest body fluid to the tumor lesion. The presence of *TP53* p. R342* in all analysed salivary samples might also indicate an increased risk of late locoregional recurrence. Indeed, we observed a suspected emi-tongue lesion that was not clinically established because of the patient's death. However, the detection of either residual tumor cells or pre-malignant lesions by *TP53* mutation analysis remains a challenge because of the difficulty in identifying a specific clinically relevant VAF cut-off. To overcome the limitations of a *TP53*-based analysis, we looked for additional biomarkers, such as circulating miRNAs. Indeed, a miRNA signature able to predict poor outcome in patients with HNSCC in tissues and liquid biopsy samples was assessed [9]. Circulating levels of miR 21–3p, miR 21–5p, miR 96–5p, and miR 429 were evaluated in both serum and salivary swabs by dPCR. Notably, we found that miRNA signature expression was increased in salivary swabs collected immediately after radiotherapy (T3) and peaked up 2 months before clinical evidence of metastasis development (T4), when compared to the sample collected at T0 (Fig. 1E and F). We did not find a similar and concomitant increase of miRNA signature in serum; thereby supporting the hypothesis that biomarker modulation can be detected earlier in saliva than in serum, as saliva is the closest body fluid to the localization of the primary tumor (Fig. 1E). Of note, the increased expression of the miRNAs signature in body fluids matched with the higher VAF of *TP53* p. R342*. As for miRNA-429, which was previously recognized as an exclusive biomarker of the salivary matrix, being absent in serum samples, its expression correlated with the trend observed for *TP53* p. R342* circulating mutation (Fig. 1F).

Collectively, we document that: (i) a combined liquid biopsy approach is feasible; and (ii) it allows accurate detection of both genetic and non-genetic alterations which cumulatively foster and predict either local tumor relapse or metastasis. Prospectively, liquid biopsy might hold important prognostic and therapeutic implications for HNSCC patients.
